# SMAD2 Inactivation Inhibits CLDN6 Methylation to Suppress Migration and Invasion of Breast Cancer Cells

**DOI:** 10.3390/ijms18091863

**Published:** 2017-08-30

**Authors:** Yan Lu, Liping Wang, Hairi Li, Yanru Li, Yang Ruan, Dongjing Lin, Minlan Yang, Xiangshu Jin, Yantong Guo, Xiaoli Zhang, Chengshi Quan

**Affiliations:** 1 The Key Laboratory of Pathobiology, Ministry of Education, College of Basic Medical Sciences, Jilin University, Changchun 130021, China; luyanw87@gmail.com (Y.L.); wlp1008@qmu.edu.cn (L.W.); hairili@ucsd.edu (H.L.); liyr@jlu.edu.cn (Y.L.); dynee@jlu.edu.cn (Y.R.); luyan13@mails.jlu.edu.cn (D.L.); yangml14@mails.jlu.edu.cn (M.Y.); jinxs14@mails.jlu.edu.cn (X.J.); guoyt16@mails.jlu.edu.cn (Y.G.); xiaoli16@mails.jlu.edu.cn (X.Z.); 2 Clinical Pathology Research Center, Department of Pathobiology, Qiqihar Medical University, Qiqihaer 161006, China; 3 Department of Cellular and Molecular Medicine, University of California, San Diego, La Jolla, CA 92093-0651, USA; lihairi@yahoo.com

**Keywords:** CLDN6, DNMT1, methylation, SMAD2, breast cancer

## Abstract

The downregulation of tight junction protein CLDN6 promotes breast cancer cell migration and invasion; however, the exact mechanism underlying CLDN6 downregulation remains unclear. CLDN6 silence is associated with DNA methyltransferase 1 (DNMT1) mediated DNA methylation, and DNMT1 is regulated by the transforming growth factor beta (TGFβ)/SMAD pathway. Therefore, we hypothesized that TGFβ/SMAD pathway, specifically SMAD2, may play a critical role for CLDN6 downregulation through DNA methyltransferase 1 (DNMT1) mediated DNA methylation. To test this hypothesis, we blocked the SMAD2 pathway with SB431542 in two human breast cancer cell lines (MCF-7 and SKBR-3). Our results showed that treatment with SB431542 led to a decrease of DNMT1 expression and the binding activity for CLDN6 promoter. The methylation level of CLDN6 promoter was decreased, and simultaneously CLDN6 protein expression increased. Upregulation of CLDN6 inhibited epithelial to mesenchymal transition (EMT) and reduced the migration and invasion ability of both MCF-7 and SKBR-3 cells. Furthermore, knocked down of CLDN6 abolished SB431542 effects on suppression of EMT associated gene expression and inhibition of migration and invasion. Thus, we demonstrated that the downregulation of CLDN6 is regulated through promoter methylation by DNMT1, which depends on the SMAD2 pathway, and that CLDN6 is a key regulator in the SMAD2/DNMT1/CLDN6 pathway to inhibit EMT, migration and invasion of breast cancer cells.

## 1. Introduction

Claudins (CLDNs) are small transmembrane proteins, and 27 members have been identified for this protein family [1,2,3]. Claudin 6 (CLDN6) is a component of tight junctions (TJs), which maintain cell–cell junctions in epithelial cell sheets. In previous studies, we demonstrated that CLDN6 mitigated the malignant phenotype of MCF-7 breast cancer cells, and the expression of CLDN6 was undetectable or at low levels in human breast cancer cells [4]. Similarly, silencing of CLDN6 enhanced migration ability of the human breast epithelium cell line HBL-100 [5]. Epithelial to mesenchymal transition (EMT) is one of the mechanisms of tumor migration and invasion [6,7,8]. During the initial stage of EMT, the expression of epithelial genes is suppressed, whereas mesenchymal marker expression is increased [9]. We believe that CLDN6 may inhibit migration and invasion of cancer cells via EMT suppression. However, the exact mechanism underlying CLDN6 downregulation remains unclear.

DNA methyltransferases (DNMTs) lead to ectopic methylation and gene silencing. De novo methylation is established by DNA methyltransferase 3 alpha (DNMT3A) and DNA methyltransferase 3 beta (DNMT3B) in early development. Once established, the methylation patterns are faithfully maintained by DNA methyltransferase 1 (DNMT1) [10,11]. Our previous study showed that CLDN6 silencing in breast cancer cells was associated with DNMT1 mediated DNA methylation [12]. Furthermore, DNMT1 is regulated by transforming growth factor (TGF)β/SMADs pathway. In this pathway, TGFβ regulates the transcription of downstream genes via the translocation of SMAD2/3 into the nucleus and then the formation of transcriptional complexes [13]. Therefore, we hypothesize that SMAD2 plays a critical role in regulating the expression and activity of DNMT1, the upregulation of which leads to CLND6 promoter hypermethylation and downregulation of CLDN6 expression, which promote EMT and enhancesmigration and invasion ability of breast cancer cells. Our results presented in current study provided evidence in support of the above hypothesis.

## 2. Results

### 2.1. SMAD2 Signaling Suppresses CLDN6 in MCF-7 and SKBR-3 Cells

**Figure 1:**
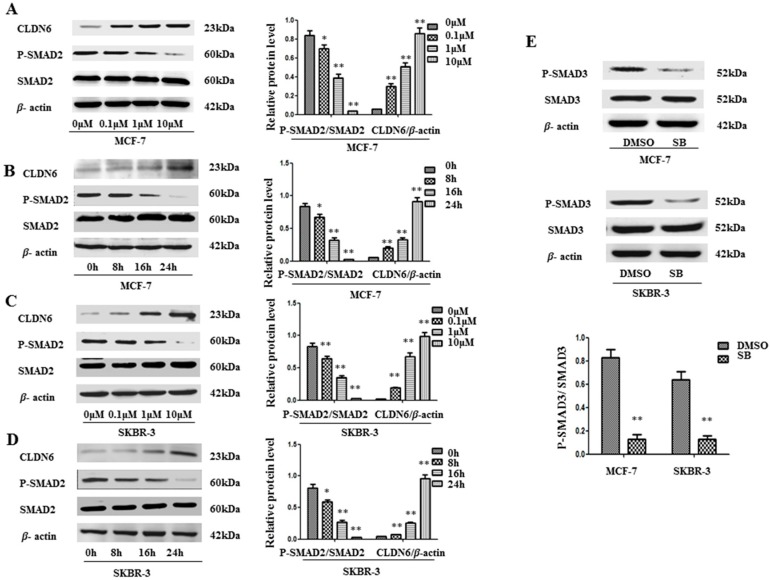
SB431542 inactivated SMAD2 signaling, and suppressed CLDN6 in MCF-7 and SKBR-3 cells. (**A**–**D**) SB431542 downregulated P-SMAD2 expression in a time- and dose-dependent manner. SB431542 treatment increased CLDN6 and decreased P-SMAD2. (**A**,**C**) MCF-7 and SKBR-3 cells were incubated with SB431542 for 24 h at the indicated concentrations. (**B**,**D**) MCF-7 and SKBR-3 cells were incubated with SB431542 at 10 μM for the indicated time (**E**) P-SMAD3 expression was decreased by SB431542 treatment. Immunoblot analysis was used to determine the expression of P-SMAD2, CLDN6, and P-SMAD3. Results of densitometry analysis of relative expression levels of P-SMAD2 and P-SMAD3 after normalization to SMAD2 and SMAD3 and CLDN6 expression levels after normalization to loading control β-actin are presented (* *p* < 0.05 and ** *p* < 0.01 are considered statistically significant and highly statistically significant, respectively). Bars represent mean ± SE (*n* = 3).

To understand the mechanisms by which CLDN6 is regulated, we used SB431542, which efficiently inhibits the activity of activated activin receptor-like kinase 4 (ALK4), ALK5, and ALK7 to phosphorylate SMAD2, to inhibit the activity of SMAD2 [14,15]. As shown in Figure 1, dose dependent and time dependent effects of SB431542 on the reduction of P-SMAD2 were observed. In both MCF-7 and SKBR-3 cells, the optimal concentration of SB431542 was 10 µM (Figure 1A,C), and the optimal time was 24 h (Figure 1B,D). In all of the following experiments, 10 µM SB431542 was used for 24 h. Simultaneously, substantial increases of CLDN6 protein was observed in a dose and time dependent manner, which is very well correlated with the decrease of SMAD2 phosphorylation levels (Figure 1A–D). We also examined the phosphorylated SMAD3 proteins in the two cell lines by Western blot analysis (Figure 1E). Similarly, P-SMAD3 was also considerably downregulated by SB431542. These results suggest that SMAD pathways regulate the expression of CLDN6, and inactivation of SMAD2/3 proteins restore CLDN6 expression in breast cancer cells.

### 2.2. SMAD2 Downregulated CLDN6 through DNA Methyltransferase 1 (DNMT1) Mediated Methylation

**Figure 2:**
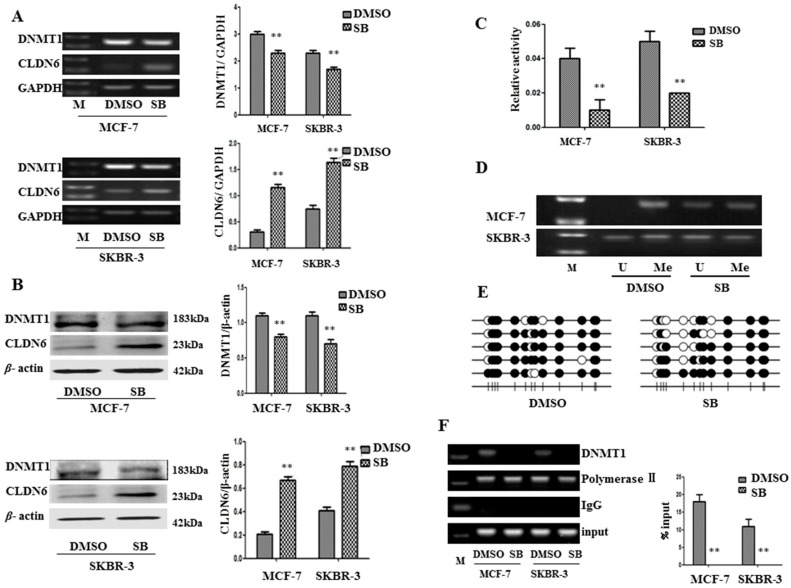
DNA methyltransferase 1 (DNMT1)-mediated upregulation of CLDN6 expression by SB431542. (**A**,**B**) Real-time polymerase chain reaction (RT-PCR) and immunoblot analysis of DNMT1 and CLDN6, and densitometric analysis of relative gene expression levels after normalization to loading controls GAPDH and β-actin are presented. (**C**) DNMT1 activity assays. (**D**) Methylation-specific PCR (MSP) analysis of CpG island of CLDN6 promoter using bisulfite-treated genomic DNA isolated from MCF-7 and SKBR-3 cells. (**E**) CpG island methylation within the CLDN6 promoter region was measured by bisulfite sequencing in SKBR-3 cells. “Me” stands for methylated, and “U” stands for unmethylated. (**F**) Chromatin immunoprecipitation-polymerase chain reaction (ChIP-PCR) assay to detect the binding of DNMT1 to the promoter of CLDN6 (** *p* < 0.01). The lane “M” stands for marker; bars represent mean ± SE (*n* = 3).

In order to determine whether SMAD2 suppresses CLDN6 expression through DNMT1 mediated methylation, we measured the changes in DNMT1 levels and activity and CLDN6 expression in MCF-7 and SKBR-3 cells after SB431542 treatment. As expected, addition of SB431542 to these cells decreased the expression of DNMT1 and increased the expression of CLDN6 at both mRNA and protein levels (Figure 2A,B), and the activity of DNMT1 was also decreased by 63.76 and 57.14% in MCF-7 and SKBR-3 cells, respectively (Figure 2C). To determine whether SB431542 altered the methylation status of the CLDN6 promoter, we performed methylation-specific PCR (MSP) analysis and demonstrated a decrease in methylation specific regions of CLDN6 promoter in MCF-7 and SKBR-3 cells treated with SB431542 (Figure 2D). We also measured CpG island methylation within the CLDN6 promoter region −300 bp~+200 bp by bisulfite sequencing PCR and found that DNA demethylation occurred at the CLDN6 promoter following SB431542 treatment (Figure 2E). To validate the impact of SB431542 on CLDN6 promoter demethylation by targeting DNMT1, we performed the chromatin immunoprecipitation (ChIP) assay to detect changes in the binding of DNMT1 to the CLDN6 promoter after treatment with SB431542. Consistent with our previous observation [12], DNMT1 bound to the CLDN6 promoter and enhanced its methylation. More importantly, SB431542 treatment substantially reduced the binding ability of DNMT1 to the CLDN6 promoter (Figure 2F). Thus, our results suggest that SMAD2 may regulate CLDN6 through DNMT1 mediated methylation.

### 2.3. Inactivation of SMAD2 Suppressed Epithelial to Mesenchymal Transition (EMT) and Inhibited Migration and Invasion of Breast Cancer Cells

**Figure 3:**
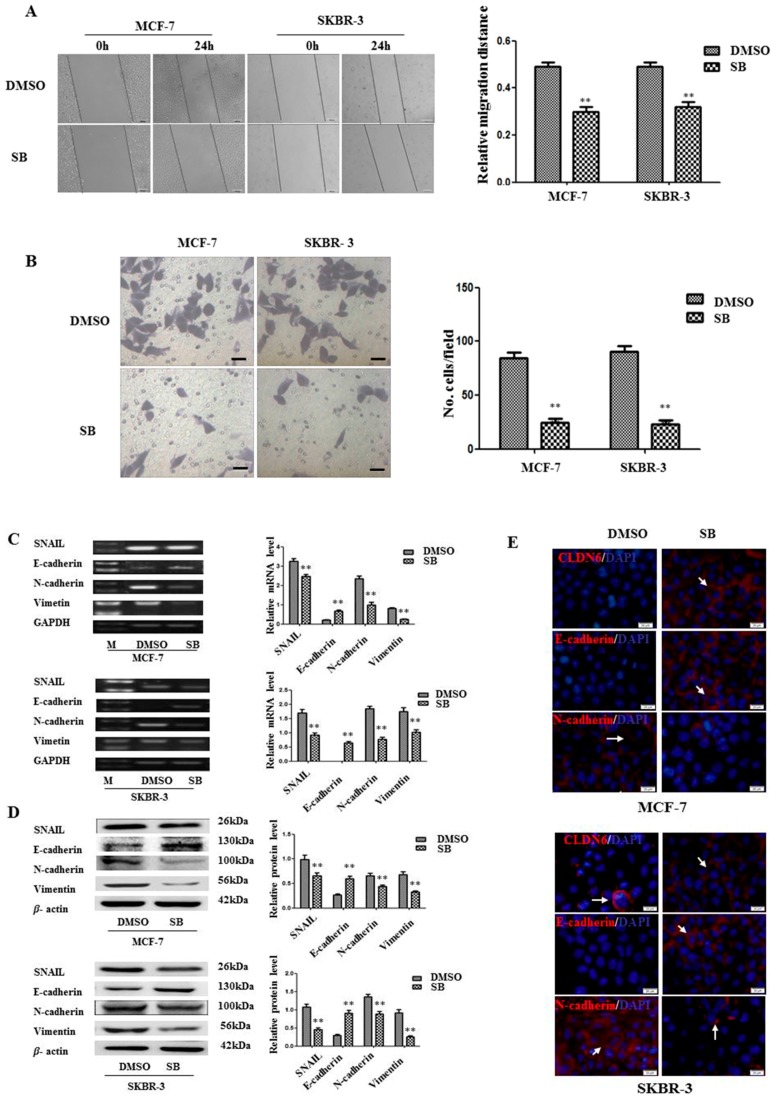
DNMT1-regulated CLDN6 expression inhibited tumor cell migration and invasion by blocking epithelial to mesenchymal transition (EMT). (**A**) Representative light microscope images of wound-healing assays for MCF-7 and SKBR-3 cells after treatment with or without SB431542 to evaluate their migration rate into the cell-free area (bar, 200 μm). (**B**) Matrigel invasion assay. Cells that invaded through the Matrigel were stained with Giemsa and counted. All results are presented as the average of cells counted in 10 fields per condition (bar, 50 μm). (**C**) RT-PCR; and (**D**) Western blotting analysis was used to determine the expression of EMT-related genes (SNAIL, E-cadherin, N-cadherin, and vimentin) in MCF-7 and SKBR-3 cells. Results of densitometric analysis of relative expression levels after normalization to loading control GAPDH or β-actin are presented. (**E**) Immunofluorescence analysis of the expression levels of EMT markers in MCF-7 and SKBR-3 cells. Representative immunofluorescence images (200×) generated using anti-E-cadherin, anti-N-cadherin, and CLDN6 primary and FITC-conjugated secondary antibodies in the breast cancer cells. Blue, cell nuclei were stained with 4,6-diamidino-2-phenylindole. Localization of E-cadherin, N-cadherin, and CLDN6 (red) at the cell–cell junctions is indicated by white arrows (bar, 20 μm). ** *p* < 0.01. Bars represent mean ± SE (*n* = 3).

To explore the outcome of SMAD2 inactivation on CLDN6 regulation, we first detected cellular morphological alterations after treatment with SB431542. It is known that CLDN6 participates in cellular TJ formation and TJ stability [16]. Thus, downregulation of CLDN6 could lead to a more invasive phenotype in cancer cells. Indeed, after SB431542 treatment, cell migration and invasion were examined and significant inhibition of both migration and invasion were found in SB431542 treated breast cancer cells; 38.54 and 35.92% migration reduction and 72.84 and 77.01% cells invasion inhibition were observed for MCF-7 and SKBR-3 cells, respectively (Figure 3A,B). To investigate the mechanism by which migration and invasion were inhibited, we examined the changes in EMT associated genes in MCF-7 and SKBR-3 cells after treatment with SB431542. SNAIL, vimentin, and N-cadherin were found to be downregulated, while E-cadherin was upregulated at both mRNA (Figure 3C) and protein (Figure 3D,E) levels following SB431542 treatment. Therefore, inactivation of SMAD2 upregulated CLDN6 suppressed EMT, and subsequently inhibited the migration and invasion of breast cancer cells.

### 2.4. Deregulation of CLND6 Is Necessary for SMAD2 Induced EMT and Tumor Cell Migration and Invasion

**Figure 4:**
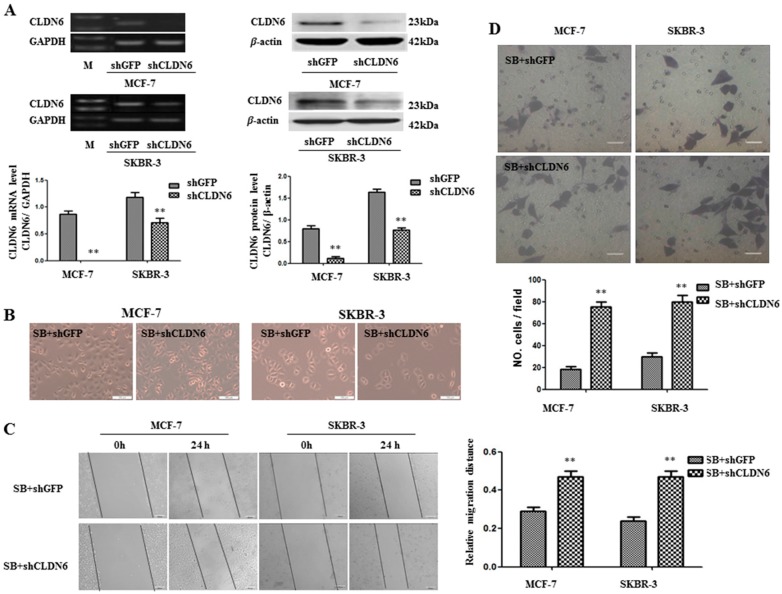

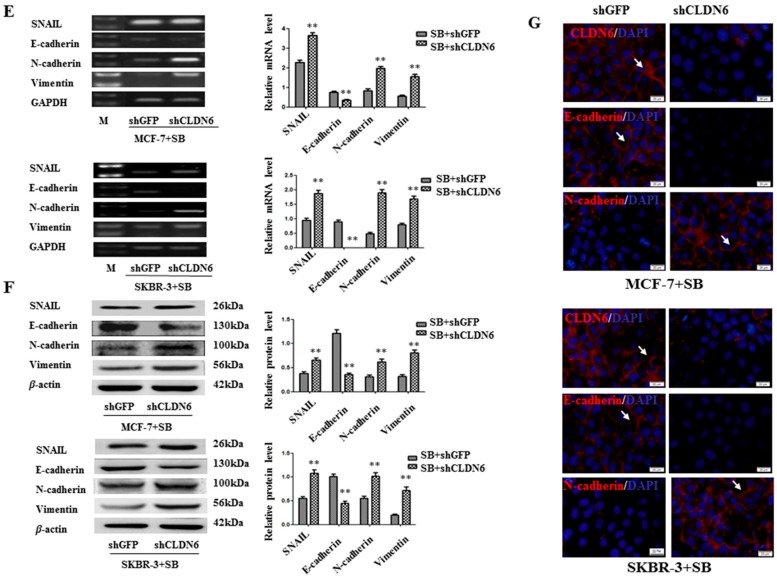
CLDN6 was required to inhibit EMT and tumor migration and invasion. Cells were stimulated with 10 µM SB431542 for 24 h. (**A**) RT-PCR and immunoblot expression analyses of CLDN6 in MCF-7-shGFP, MCF-7-shCLDN6, SKBR-3-shGFP, and SKBR-3-shCLDN6 cells, and densitometric analysis of relative expression levels after normalization to loading control GAPDH or β-actin are presented. (**B**) The effect of knocking down CLDN6 on cell morphology of SB431542 treated MCF-7 and SKBR-3 cells (bar, 100 μm). (**C**) Representative light microscope images of wound-healing assays for MCF-7 and SKBR-3 cells treated with SB431542 to evaluate the impact of CLDN6 on their migration rate into the cell-free area (bar, 200 μm). (**D**) Matrigel invasion assay. Cells that invaded the Matrigel were stained with Giemsa and counted. All results are presented as the average of cells counted in 10 fields per condition (bar, 50 μm). Results of: (**E**) RT-PCR; and (**F**) Western blotting analyses to determine the impact of CLDN6 on the expression of EMT-related genes (SNAIL, E-cadherin, N-cadherin, and vimentin) in MCF-7and SKBR-3 cells treated with SB431542, and densitometric analysis of relative gene expression levels after normalization to the loading control GAPDH or β-actin are presented. (**G**) Immunofluorescence analysis to evaluate the impact of CLDN6 on the expression levels of EMT markers in MCF-7 and SKBR-3 cells treated with SB431542. Representative immunofluorescence images (200×) generated using anti-E-cadherin, anti-N-cadherin, and CLDN6 primary and FITC-conjugated secondary antibodies in the breast cancer cells. Blue, cell nuclei were stained with 4,6-diamidino-2-phenylindole. Localization of E-cadherin, N-cadherin, and CLDN6 (red) at the cell–cell junctions is indicated by white arrows (bar, 20 μm). ** *p* < 0.01. Bars represent mean ± SE (*n* = 3).

The observations that SB431542 upregulated CLDN6, inhibited EMT, and suppressed migration and invasion led us to ask whether SB431542 induced cellular phenotype changes were mediated by CLDN6. Knocking down CLDN6 by shRNA (Figure 4A) leads to morphological change of MCF-7 cells into a spindle-shaped cells, but morphological change is not obvious in SKBR-3 cell line (Figure 4B). Furthermore, knocking down CLDN6 abrogated SB431542 mediated inhibition of migration and invasion, as measured by wound healing and transwell migration assays (Figure 4C,D), as well as suppressed the expression of EMT associated genes (Figure 4E–G). Thus, CLDN6 is the key regulator in the SMAD2/DNMT1/CLDN6 pathway to inhibit EMT and migration and invasion of breast cancer cells.

## 3. Discussion

In earlier studies, we found that CLDN6 overexpression inhibited the migration and invasion of MCF-7 cells in vitro [4], while the expression of CLDN6 was undetectable or low in several human cancer cells [17,18]. The exact mechanism leading to CLDN6 downregulation, however, remains unclear. We and others also demonstrated that the silencing of CLDN6 was linked to DNMT1 mediated DNA methylation [12], and that DNMT1was regulated by TGFβ/SMADs pathways [19,20]. DNMT1 possesses a unique capability of identifying the hemimethylated portion of newly replicated DNA. This feature might explain why DNMT1 mediated methylation could be an epigenetic mechanism maintaining the status quo [21,22]. It has been shown that DNMT1 maintains DNA methylation and results in the silencing of tumor suppressor genes [23,24]. Biniszkiewicz and colleagues reported that increased DNMT1 activity led to hypermethylation of CpG islands both in vivo and in vitro [25]. Hypermethylation of CpG islands is also frequently observed in cancer and has been shown to be involved in the silencing of tumor suppressor genes [26]. A recent report showed that DNMT1 plays a key role in the regulation of CLDN4 and CLDN7 by means of the SMAD signaling pathway [19]. Expression of CLDNs epithelial cells is a dynamic equilibrium pattern, high expression of some CLDN member proteins leads to a low expression of other members of the CLDN family [27]. We also found that, compared to immortalized breast epithelial cell line HBL-100, SMAD2 showed a higher expression in breast cancer cells, whereas CLDN6 had a lower expression level in these cells.

In the current study, we aimed to explore the mechanism by which upstream signaling downregulates CLDN6 during mammary cancer progression. The two different breast cancer cell lines used, MCF-7 and SKBR-3, are luminal subtypes of metastatic adenocarcinoma. The MCF-7 cell line is estrogen receptor (ER)^+^, progesterone receptor (PR)^+^, and ERBB2/HER2^−^, while the SKBR-3 cell line is ER^−^, PR^−^, and ERBB2/HER2^+^ [28]. After treating the two breast cancer cell lines with SB431542, a TGFβ type I receptor inhibitor that specifically inhibits SMAD2 and SMAD3 phosphorylation [14,29,30], which leads to down regulation of DNMT1 and upregulation of CLDN6. Furthermore, both DNMT1 enzyme activity and capacity to CLNDN6 promoter were reduced, and simultaneously, the CLDN6 promoter methylation status also decreased significantly. We also measured CpG island methylation within the CLDN6 promoter region by bisulfite sequencing and found that DNA demethylation occurred at the CLDN6 promoter following SB431542 treatment. Our data demonstrated that, in breast cancer cells, the low expression of CLDN6 was regulated by DNMT1 mediated methylation, which was in turn regulated by the SMAD2 pathway.

Similar to our current results, CLDN6 has been found to be silenced by methylation in esophageal squamous cell carcinoma [31]. Besides DNA methylation, CLDN6 also can be regulated by histone modification [32]. Furthermore, in our previous study, we demonstrated that DNA methylation of CLDN6 enhanced breast cancer cell migration and invasion by recruiting methyl CpG binding protein 2 (MeCP2) and deacetylating histone 3 acetylation (H3Ac) and histone 4 acetylation (H4Ac) [12]. Our current study, we showed that DNMT1 could directly regulate CLDN6 through binding to its promoter region. Papageorgis and colleagues showed that DNMT1 played a key role in the regulation of CLDN4 and CLDN7 via the SMAD2 signaling pathway and that the depletion of SMAD2 led to a significant decrease in the amount of DNMT1 bound to the promoter of the target genes, but it did not decrease the expression level of DNMT1 [19], which is different with our current results. Furthermore, SMAD pathways and many major signaling pathways, including those involving Wnt and extracellular signal–regulated kinase (ERK)/Mitogen-activated protein kinase (MAPK), have been reported to be involved in DNA methylation [20,33,34,35,36].

CLDNs are the main components of TJs, which have barrier and fence functions and maintain cell polarity, cell adhesion, and cell signal transduction [37,38], and dysfunction of TJs was found to be closely related to tumor development, e.g., CLDN6 overexpression inhibited the migration and invasion of MCF-7 cells in vitro [4]. One of the mechanisms of migration and invasion in epithelium-derived carcinoma was EMT [39] which led to the loss of epithelial cell adhesion and the induction of a mesenchymal phenotype [40,41]. Recent reports have shown that EMT, which is characterized by the loss of epithelial markers such as E-cadherin and the induction of mesenchymal markers, including vimentin, N-cadherin, and fibronectin [42], plays a pivotal role in breast cancer progression [43,44,45]. In the present study, CLDN6 expression in breast cancer cells was upregulated by SB431542 treatment, and, subsequently, the invasion and migration were suppressed, along with the upregulation of epithelial marker E-cadherin and the downregulation of mesenchymal markers SNAIL, N-cadherin, and vimentin. Thus, we demonstrated that CLDN6 inhibited invasion and migration by reversing EMT.

Consistent with our data, CLDN3, CLDN4 [46], and CLDN7 [47] have been shown to suppress EMT in ovarian carcinoma cells and lung cancer cells. However, CLDN1 suppressed E-cadherin and subsequently induced EMT in hepatocellular carcinoma cells [48]. The levels of CLDN3 and CLDN4 were frequently elevated in pancreatic adenocarcinoma, ovarian cancer, endometrioid adenocarcinoma, and prostate cancer [49,50,51,52], while CLDN7 has been found to be decreased in invasive ductal carcinomas of the breast [53]. This can be attributed to tissue and cell specificity of expression and distribution of CLDNs, which is the main reason for different TJ functions in different types of epithelial tissues [54]. Similarly, the distribution of CLDNs differs greatly in different structures within the same organization [55,56].

TGFβ was first described as an EMT inducer in normal mammary epithelial cells [57] and has been recognized as an EMT master regulator in different cell types [40]. In the current study, we demonstrated that SB431542 inhibited EMT and suppressed migration and invasion of MCF-7 and SKBR-3 cells by upregulating CLDN6 but not the direct effect of inactivation of TGFβ/SMAD pathway. To verify this assumption, we knocked down CLDN6 in SB431542-treated MCF-7 and SKBR-3 cells and found knocking down CLDN6 abrogated the inhibitory effects of SB431542 on EMT, migration and invasion of breast cancer cells, indicating CLDN6 is the key regulator downstream of the SMAD2/DNMT1/CLDN6 pathway. CLDN6 is also an epithelial marker, which was upregulated when EMT was inhibited. It may be confused whether EMT was suppressed before the upregulation of CLDN6. The results in Figure 4 demonstrated that CLDN6 was upregulated and then suppressed EMT in SB431542-treated MCF-7 and SKBR-3 cells.

In conclusion, SMAD2 downregulated CLDN6 via DNMT1 mediated DNA methylation to promote EMT, thereby accelerating the migration and invasion of breast cancer cells.

## 4. Materials and Methods

### 4.1. Cell Culture and Reagents

The human breast cancer cell lines MCF-7 and SKBR-3 were obtained from the Cell Bank of the Chinese Academy of Sciences (Shanghai, China) and maintained in DMEM (Gibco, Carlsbad, CA, USA) supplemented with 10% fetal bovine serum (Hyclone, Logan, UT, USA) and 100 units/mL penicillin and streptomycin (Invitrogen, Carlsbad, CA, USA). All the cell lines were grown in a humidified incubator at 37 °C and 5% CO_2_. SB431542 was purchased from Sigma (St. Louis, MO, USA).

### 4.2. Reverse Transcription Polymerase Chain Reaction

Total RNA was extracted from cells using TRIzol (Invitrogen) following the manufacturer’s instructions. One microgram of total RNA was subjected to reverse transcription to synthesize cDNA using Moloney murine leukemia virus reverse transcriptase (TaKaRa, Osaka, Japan) at 42 °C for 1 h, and 0.5 µg cDNA was used for PCR. SMAD2, CLDN6, DNMT1, SNAIL, E-cadherin, and N-cadherin were amplified along with glyceraldehyde-3-phosphate dehydrogenase (GAPDH) as an endogenous control following the instructions of the Premix LA Taq Kit (TaKaRa). The PCR conditions and primer sequences are shown in Table 1. After electrophoresis, the gel was imaged and analyzed by an imaging system (Syngene, Cambridge, UK).

### 4.3. Western Blotting Analysis

Western blotting analyses were performed as described previously [12]. Anti-P-SMAD2, SMAD2, P-SMAD3, SMAD3, and vimentin antibodies were purchased from Cell Signaling Technology (Beverly, MA, USA); E-cadherin and SNAIL antibodies were from Bioworld Technology (Dublin, OH, USA); and N-cadherin, DNMT1, and CLDN6 antibodies were from Abcam (Cambridge, UK). The anti-β actin antibody was obtained from Santa Cruz (Santa Cruz, CA, USA). Primary and secondary antibodies were diluted to 1:1000. The blots were stained using an ECL Western blotting system (GE, Fairfield, CT, USA).

### 4.4. Immunofluorescence

An immunofluorescence assay was performed to evaluate expression as previously described [58]. Cells were incubated with primary antibodies against CLDN6 (1:400), E-cadherin (1:400), and N-cadherin (1:250) at 4 °C overnight. The secondary antibody was Alexa Fluor 647 anti-mouse IgG (1:200 dilution; Cell Signaling Technology, Beverly, MA, USA). Cells were visualized with a fluorescence microscope (Olympus, Tokyo, Japan).

### 4.5. DNA Methylation Analysis

MSP based on bisulfite conversion was performed. Genomic DNA from cells was isolated using the DNA extraction kit (Qiagen, Valencia, CA, USA), and the methylSEQr™ Bisulfite Conversion Kit (Applied Biosystems, Grand Island, NY, USA) was used for sodium bisulfite treatment of the genomic DNA according to the manufacturer’s protocol. MSP primers were designed with the aid of the Methyl Primer Express v1.0 software (Applied Biosystems, Lincoln, CA, USA) (Table 1). PCR products were purified from 1.5% agarose gels using a Gel Extraction Kit (Qiagen) and cloned into the pGEM-T Easy vector (Promega, Madison, WI, USA). Five randomly selected clones from each sample were chosen for sequencing.

### 4.6. DNA Methyltransferase 1 (DNMT1) Activity Assays

Nuclear protein was isolated using the Nuclear Protein Extraction Kit (BSP009; GeneChem Co. Ltd., Shanghai, China). The DNMT1 Activity Assay Kit (GMS50080; GenMed, Plymouth, MN, USA) was used for detection of DNMT1 activity according to the manufacturer’s protocol. Replicates of each sample (20 μg nucleoprotein, including blank and positive control) were analyzed to validate the signal generated. The DNMT activity data were presented as OD/h/mg.

### 4.7. Wound Healing Assays

Cells (1 × 10^6^) were grown overnight in 60 mm dishes to reach confluency, and a wound was introduced using a Q tip. Images were obtained using a microscope (Olympus) at 0 and 24 h after wounding to determine the width of the wounded area. The relative migration distance (percent of recovery) was calculated as (W_0_ − W_24_)/W_0_ × 100% (W_0_ indicates wound width at 0 h, and W_24_ indicates wound with at 24 h).

### 4.8. Matrigel Invasion Assays

Matrigel invasion assays were performed using Transwells containing 8.0 μm pore membranes (Corning, Lowell, MA, USA). MCF-7 and SKBR-3 cells were placed in the upper chamber of the Transwell, and 48 h later, the chambers were washed twice with phosphate buffer solution (PBS). The filter side of the upper chamber was cleaned with a cotton swab. Next, the membrane was cut out of the insert. Cells were fixed in methanol and stained with 5% Giemsa for 30 min at room temperature.

### 4.9. Chromatin Immunoprecipitation Assay

Chromatin immunoprecipitation (ChIP) assay was performed using the EZ Magna ChIP G Chromatin Immunoprecipitation kit (Millipore, Billerica, MA, USA) using chromatin isolated from 1 × 10^6^ cells per condition, according to the manufacturer’s protocol. Antibodies included anti-DNMT1 (1:500, Cell Signaling Technology), anti-IgG (Millipore), and anti-DNA polymerase II (Millipore); anti-IgG was the negative control and anti-DNA polymerase II was the positive control.

### 4.10. Transfection With Short Hairpin RNA

Cells were transfected with short hairpin RNA (shRNA) by using Lipofectamine 2000 (Invitrogen), following the procedure recommended by the manufacturer. shRNA targeting of CLDN6 (5’-GTGCAAGGTGTACGACTCA-3’) and a negative control shRNA were purchased from GeneChem Co. Ltd.

### 4.11. Statistical Analysis

All computations were carried out using the SPSS version 19.0 for Windows (SPSS Inc., Chicago, IL, USA). Unpaired *t*-tests were performed to evaluate data for target mRNA and protein. The data are presented as means ± standard error from at least three independent experiments. *p* < 0.05 was considered statistically significant.

## Figures and Tables

**Table 1:** Details of primers used for real-time polymerase chain reaction (RT-PCR), methylation-specific PCR (MSP), and chromatin immunoprecipitation (ChIP) analyses.

Primer Name	Primer Sequence	Annealing Temp (°C)	Cycles	Length (bp)
CLDN6	TTCATCGGCAACAGCATCGT	58	35	345
	GGTTATAGAAGTCCCGGATGA			
DNMT1	GAGGAAGCTGCTAAGGACTAGTTC	56	30	141
	ACTCCACAATTTGATCACTAAATC			
SMAD2	ATTTGCTGCTCTTCTGGCTCAG	56	30	101
	ACTTGTTACCGTCTGCCTTCG			
SNAIL	GCCTAGCGAGTGGTTCTTCTG	56	30	203
	TAGGGCTGCTGGAAGGTAAA			
E-cadherin	ATTCTGGGGATTCTTGGAGG	56	30	337
	GGTCAGTATCAGCCGCTTTC			
N-cadherin	GTGCCATTAGCCAAGGGAATTCAGC	56	30	370
	GCGTTCCTGTTCCACTCATAGGAGG			
Vimentin	AGCAGG AGTCCACTGAGTACCG	56	30	200
	GTGACGAGCCATTTCCTCCTTC			
GAPDH	TGTTGCCATCAATGACCCCTT	56	25	178
	CTCCACGACGTACTCAGCG			
CLDN6 U (MSP)	TGGATGTTTGTTAGTTTGAGGT	58	35	500
	ATAACCACAACC CAAATTCACA			
CLDN6 M (MSP)	ACGTTTGTTAGTTCGAGGC	58	35	502
	ATAACCGCAACCCGAATTC			
CLDN6 (BSP)	GAGGGGTAGAGATTTTGTTTTTGA	53	30	210
	AATTAAATAAATTCCCCATATCACC			
CLDN6 (ChIP)	GCCACCTGGATGGCCGAGTC	51	40	191
	GAGGGTTCCCAATTTGCGGG			
